# Onabotulinum Toxin A Is a Viable Intervention for Bladder Neck Obstruction in Women: A Prospective Pilot Study of Patient Reported Outcomes

**DOI:** 10.1002/nau.70240

**Published:** 2026-02-18

**Authors:** Brittany Lee Roberts, Darrell Bibicheff, Elise J. B. De

**Affiliations:** ^1^ Department of Obstetrics and Gynecology Albany Medical Center Albany New York USA; ^2^ Albany Medical College Albany New York USA; ^3^ Department of Urology Albany Medical Center Albany New York USA

**Keywords:** bladder neck botox, bladder neck obstruction, chronic pelvic pain

## Abstract

**Introduction:**

Primary bladder neck obstruction (BNO) occurs when the bladder neck fails to open during voiding. The cause of BNO is not fully understood but may be related to smooth muscle hypertrophy, increased collagen deposition, or sympathetically mediated high‐tone smooth muscle of the urethra. As there is symptom overlap with other urinary pathology, diagnosis is challenging, and there are limited treatments with a paucity of data. Onabotulinum toxin A (BoNT‐A) to the bladder neck has shown improvement in symptoms caused by BNO in women in a small retrospective study. We aimed to prospectively analyze the therapeutic efficacy of BoNT‐A to the bladder neck as a treatment option for women with BNO.

**Materials and Methods:**

We performed a pilot study recruiting female patients with pelvic pain and BNO from September 2023 to July 2024. Patients were diagnosed with BNO using the urodynamic Nitti Criteria, patient symptoms of hesitancy, straining, and/or dysuria, and cystoscopic evidence suggesting BNO. Patients were assessed prior to BoNT‐A injection to the bladder neck and 4‐6 weeks post‐procedure. The procedure consisted of 100 units of BoNT‐A reconstituted to 2 mL of Marcaine or saline with 0.5 mL injected cystoscopically at 10, 2, 5, and 7 o'clock in the bladder neck. The primary outcome was the change in the Female Genitourinary Pain Index Scale (Female GUPI, scores range from 0 to 44, lower scores are better). Secondary outcomes pre‐procedure versus post‐procedure included the Pelvic Floor Distress Index‐20 (PFDI‐20, scores range from 0 to 300, lower scores are better), a pain visual analogue scale (VAS, ranging from 0‐no pain to 10‐worst pain), and post‐void residual (PVR) volumes. The Global Response Assessment (GRA, −3 to +3, +3 better) was included post‐procedure. Data were analyzed using descriptive statistics, and outcomes were compared using the Wilcoxon signed‐rank test.

**Results:**

Twenty‐two female patients with BNO were recruited to our study. Patients had significant improvement in the Female GUPI with a decrease in scores from a median of 34.5 (IQR 31−36) pre‐bladder neck BoNT‐A to 26 (20.3‐29.8) post‐procedure (*p* = 0.002). The Pain, Urination, and Quality of Life subscales of the Female GUPI all demonstrated significant improvement (all *p* < 0.05). Median improvement on the GRA was 1.4 (SD 1.4). Compared to baseline, there was improvement in the Urinary Distress Index‐6 (UDI‐6) subscale (*p* = 0.012) but not in overall PFDI‐20 total scores, which includes prolapse and bowel symptoms in addition to the UDI‐6 (*p* = 0.161). The median PVR prior to treatment was 126 mL (IQR 50−193), and after treatment decreased to 28 (14−59) (*p* = < 0.001).

**Conclusions:**

BNO in women encounters limited therapeutic options. BoNT‐A to the bladder neck may be considered. BoNT‐A to the bladder neck demonstrated improvement in pain, lower urinary tract symptoms (LUTS), and PVR volumes in those with refractory BNO.

## 1 Introduction

Primary bladder neck obstruction (BNO) is a condition in which the bladder neck fails to open normally during the voiding phase of urination, resulting in increased internal urethral sphincter activity or obstruction of urinary flow in the absence of another anatomic obstruction [1]. The cause of BNO is not fully understood. Theories include smooth muscle hypertrophy or increased collagen deposition, or high‐tone in the smooth muscle of the urethra, causing rigidity of the bladder neck. Sympathetic nervous system dysfunction has been suggested as an etiology in the setting of an increased number of α‐adrenergic receptors [2, 3, 4] or small fiber neuropathy. The prevalence of BNO ranges between 5% and 11% in young women with voiding dysfunction, and women often present with lower urinary tract symptoms (LUTS) [5]. LUTS associated with BNO include hesitancy, intermittency, straining, dysuria, frequency, urgency, incomplete emptying, and urinary retention. BNO is diagnosed based on high voiding pressure and low urine flow, in addition to fluoroscopic evidence of incomplete or closed bladder neck with voiding. Cystoscopic findings that may be suggestive of BNO include bladder trabeculations or a tight bladder neck.

Treatment options for BNO in women are limited but include expectant management, pelvic floor physical therapy, pharmacotherapy, and surgical interventions. Alpha‐blockers can improve symptoms, flow rates, and post‐void residual volumes in up to 70% of women with BNO, however compliance rates are low due to side effects [6]. Transurethral incision of the bladder neck is the standard surgical intervention for BNO [7]. Although effective, risks of the procedure include urethral or bladder neck stricture and stress urinary incontinence (SUI) [8, 9]. Onabotulinum toxin A (BoNT‐A) to the bladder neck is an emerging therapy for the treatment of BNO. BoNT‐A to the bladder neck has been shown to improve pelvic pain and refractory hesitancy in women with BNO [10, 11]. Available literature is comprised of studies with small sample sizes or retrospective data. Limited data exist prospectively investigating the effect of BoNT‐A on BNO in women. We aimed to investigate prospectively the therapeutic efficacy of BoNT‐A as a treatment for women with BNO. We hypothesize that BoNT‐A will improve symptoms of hesitancy, pain, incomplete emptying, frequency, and urgency in women with BNO.

## 2 Materials and Methods

This study was reviewed and approved by the Institutional Internal Review Board. From September 2023 to July 2024, women with symptoms of hesitancy, intermittency, straining, dysuria, incomplete emptying, and urinary retention were evaluated for BNO. Urodynamic testing was performed according to the International Continence Society Good Urodynamics Practice Standards. Patients were given privacy during the void, with the lights off, water running, and the examiner out of view behind the curtain or outside the door. If the patient could not void on the cushioned Sonesta chair under fluoroscopy, they were moved to a plastic commode with the catheters in place, adjusting tranducer height. We defined BNO either (1) according to the Nitti Criteria [12] as obstructive parameters (detrusor pressure over 20 and flow under 10) referable to the bladder neck by fluoroscopy on video urodynamics or, if imaging not captured during flow then by the electromyography (EMG) remaining quiet during bladder contraction or (2) no bladder contraction mounted during UDS (e.g., shy bladder) but presence of cystoscopic evidence of obstruction, for example, trabeculation and tight bladder neck, and symptoms suggestive of BNO.

Patients were included in our prospective pilot study if they met the above criteria for BNO and failed conventional therapy (physical therapy and/or alpha blockers), were female sex (any gender if born with female anatomy and gender affirmation surgery had not yet occurred), over 18 years of age, able to provide informed consent, and naïve to BoNT‐A to the bladder neck. Patients were defined as BoNT‐A naïve if they had never had BoNT‐A to the bladder neck or had not had BoNT‐A to the bladder neck within the last 8 months. Patients were excluded if they had an allergy to BoNT‐A, had a pre‐existing neurologic or neuromuscular condition that prohibited them from receiving BoNT‐A or confounded the diagnosis of BNO, or if they were pregnant.

Eligible patients were offered participation, and written informed consent was obtained. Participants completed baseline data including: age, body mass index (BMI), validated questionnaires including the Female Genitourinary Pain Index (Female‐GUPI) [13], Pelvic Floor Distress Index; ‐20 (PFDI‐20) [14] (made up of the Pelvic Organ Prolapse Distress Inventory‐6 [POPDI‐6]) [15], Colorectal‐anal Distress Inventory‐8 (CRADI‐8) [15], Urinary Distress Inventory‐6 (UDI‐6) [16]), and pain visual analog scale (VAS). Post‐void residual (PVR) volumes were obtained pre and post‐procedure. The method of administration of BoNT‐A to the bladder neck has been previously published [11, 17]. Postoperative evaluation occurred at 4 weeks. If the patient had a urinary tract infection at the 4 week visit, there was an additional visit 6 weeks post‐procedure for repeat evaluation and data collection. All questionnaires and PVR measurements were completed prior to and 4−6 weeks after the procedure. The therapeutic response varies with the individual, but typically, patients have repeat treatments every 3 months. The post‐procedure visit also included the Global Response Assessment (GRA), which rates improvement on a Likert scale from −3 (markedly worse) to +3 (markedly improved). They were then asked to classify *which* symptoms were better, including: difficulty starting stream, pain with urination, urethral burning, frequency, urgency, feeling of incomplete emptying, or other. Lastly, patients were asked if they had a flare (temporary worsening of symptoms as BoNT‐A took hold) after BoNT‐A to the bladder neck.

The primary outcome was the change in the Female GUPI total scores. The Female GUPI is a validated questionnaire with three subscales: Pain, Urination, and Quality of Life. Scores range from 0 to 44, with higher scores indicating more severe symptoms. Secondary outcomes included the GRA, the PFDI‐20 (POPDI‐6, CRADI‐8, UDI‐6), Pain VAS, and PVR. The GRA ranges from −3 to +3 (+3 better). The PFDI‐20 scores range from 0 to 300, with subscales of POPDI‐6, CRADI‐8, and UDI‐6; higher scores indicate more severe symptoms. The pain VAS ranged from 0 to 10; 0 indicating no pain and 10 indicating the worst pain ever. Analysis was performed using SPSS version 20. Descriptive statistics were used to analyze baseline demographics and symptom evaluation post‐bladder neck BoNT‐A. The Wilcoxon signed‐rank test was used to compare primary and secondary outcomes from pre and post‐bladder neck BoNT‐A. Statistical significance was at *p* < 0.05. As this was a pilot study, no power analysis was performed. Recruitment was continued until a convenience sample of 22 was reached.

## 3 Results

A total of 22 patients met the inclusion criteria and were recruited to our pilot study. Of the 22 patients included, all performed pre and post‐procedure surveys and PVR assessments. The average age of participants was 47 ± 16.5 years, and the average BMI was 27.1 ± 7.5 kg/m [2]. Thirteen patients had tried an alpha blocker prior to BoNT‐A. Four of these experienced orthostasis and had to discontinue the alpha blocker. An additional four had pre‐existing orthostasis, prohibiting trial. Small fiber neuropathy was present in eight of the patients. Six participants received BoNT‐A to the bladder neck as the only treatment site, and 13 participants received BoNT‐A to the bladder neck and pelvic floor muscles. Two participants received BoNT‐A to the bladder neck and external urethral sphincter, one participant received BoNT‐A to the vulva, and no participants received BoNT‐A to the bladder detrusor (Table 1).

**Table 1:** Baseline characteristics.

Population characteristics	*N* = 22
Age (mean ± SD)	47.0 ± 16.5
BMI (kg/m^2^) (mean ± SD)	27.1 ± 7.5
*Procedures performed*	
Botox at BN (*N*)	6
Botox at BN and EUS (*N*)	2
Botox at BN and detrusor (*N*)	0
Botox to BN and PFM (*N*)	13
Botox to BN and vulva (*N*)	1

*Note:* Data are reported as Mean ± standard deviation or number of patients receiving respective treatments.

Abbreviations: BMI, body mass index; BN, bladder neck; EUS, external urethral sphincter; PFM, pelvic floor muscles.

The Female GUPI total scores improved after BoNT‐A injections to the bladder neck with a median of 34.5 (IQR 31−36) versus 26 (20.3−29.8); *p* = 0.002 (Table 2). The minimal clinical important difference for the GUPI (including use in female patients) is a 7‐point reduction. The difference between pre‐procedure and post‐procedure reached this difference [18]. Patients reported significant improvements in all subscales of the Female GUPI. The pain subscale decreased from a median of 16.5 (IQR 14−19) to 12.5 (9−20) (*p* = 0.02), urination subscale improved from a median 8 (IQR 6.3−9) to 5.5 (3−7.4) (*p* < 0.001), and quality of life subscale decreased from a median of 11 (IQR 9.3−12) to 7.5 (5−10) (*p* = 0.004). Global improvement reported on the GRA was median 1.5 (SD 1.4). The UDI‐6 subscale of the PFDI‐20 score improved from a median of 54.2 (IQR 45.8−79.2) to 39.6 (26.0–45.8) (*p* = 0.012). Total PFDI‐20, POPDI‐6, and CRADI‐8 scores did not significantly change from baseline, as expected (all *p* = NS). The pain VAS change did not reach significance (median of 6.5 (IQR 5−8) to 5 (3.1−7), *p* = 0.05). Patients' PVR decreased from a median of 126 mL (IQR 50−193 mL) pre‐procedure to 28 mL (14−59 mL) post‐procedure (*p* < 0.001).

**Table 2:** Comparison of Outcomes Pre and Post‐Bladder Neck Botox.

	Pre‐bladder neck botox *N* = 22	Post‐bladder neck botox *N* = 22	*p* value
	Baseline	4 Week follow‐up	
Female genitourinary pain index total	34.5 (31−36)	26 (20.3−29.8)	0.002
Pain subscale	16.5 (14−19)	12.5 (9−20)	0.02
Urination subscale	8 (6.3−9)	5.5 (3−7.4)	< 0.001
Quality of life subscale	11 (9.3−12)	7.5 (5−10)	0.004
PFDI‐20 summary score	50.3 (35.6−57.8)	116.3 (90.4−155.0)	0.2
POPDI‐6 scale score	46.9 (26.0−55.7)	33.3 (22.9−45.8)	0.1
CRADI‐8 scale score	25 (11.7−47.7)	23.4 (10.2−42.9)	0.4
UDI‐6 scale score	54.2 (45.8−79.2)	39.6 (26.0−45.8)	0.012
Pain scale (0−10)	6.5 (5−8)	5.0 (3.1−7)	0.05
PVR (mL)	126 (50−193)	28 (14−59)	< 0.001

*Note:* All data are reported as median (IQR).

Abbreviations: CRADI‐8, Colorectal‐Anal Distress Inventory‐8; EQ‐5D, EuroQol‐5 Dimension; GRA, Global Response Assessment; PFDI‐20, Pelvic Floor Distress Inventory‐20; POPDI‐6, Pelvic Organ Prolapse Distress Inventory‐6; UDI‐6, Urinary Distress Inventory‐6.

Most patients reported at least some symptom improvement post‐procedure (68.2%), categorized as patients reporting improvement (+1 = slightly to +3 = markedly) On a checklist of individual symptoms, five patients reported improvement in difficulty starting stream (22.7%), nine improved frequency (40.9%), eight improved urgency (36.4%), and eight indicated improved pain with urination (36.4%). Of note, 14/22 (63.6%) reported a temporary worsening of symptoms (flare) after the procedure. Overall, 12/22 (55%) of patients saw enough benefit to return for a repeat procedure. Among participants, 16/22 (72.7%) reported any new or worsening LUTS within the follow‐up window; the most common was difficulty initiating the urinary stream (9/22, 40.9%). Dysuria‐type symptoms were less frequent: pain with urination in 3/22 (13.6%) and urethral burning in 1/22 (4.5%). Frequency and urgency each occurred in 4/22 (18.2%), and a sensation of incomplete emptying in 6/22 (27.3%) (Table 3). Most symptoms were transient and managed conservatively; no hospitalizations occurred.

**Table 3:** Symptom evaluation post‐bladder neck botox at 4 weeks post‐procedure.

	*N* = 22 (%)
Overall, I feel my symptoms improved (scale −3 to +3, +1 to +3 is improvement)	15 (68.2)
Which symptoms improved?*	
Difficulty starting stream	5 (22.7)
Pain with urination	8 (36.4)
Urethral burning	4 (18.2)
Frequency	9 (40.9)
Urgency	8 (36.4)
Feeling of incomplete emptying	3 (13.6)
I had a temporary flare of symptoms after a Botox injection	14 (63.6)

*Note:* Data presented as *N* (%) *Total greater than 22, as patients could report more than one symptom improving.

Outcomes were also compared to urodynamic characteristics. Improvement after BoNT‐A was reported in 8/11 (73%) of patients who had BNO by pressure parameters (PdetQmax greater than 20 cm H_2_0 and a flow less than 10 mL/s), 4/6 (67%) of those with BNO by Nitti Criteria (obstructive pressure parameters and fluoroscopy showing a closed bladder neck) and 7/11 (64%) of those who met the diagnosis of BNO by inability to void (shy bladder) or straining to void on UDS in combination with cystoscopic findings and symptoms attributable to BNO.

## 4 Discussion

BoNT‐A treatment of the bladder neck improves pain and LUTS in women with BNO refractory or contraindicated to conservative management. We observed significant improvement in the Female‐GUPI total and pain, urination, and quality of life subscales, the GRA, UDI‐6, and PVR volumes after BoNT‐A treatment to the bladder neck. BNO in women is challenging to diagnose, given the common symptom of difficulty initiating stream during testing, and once diagnosed, consensus is lacking regarding the best treatment modality [10]. The patient‐reported outcomes in this prospective pilot study suggest that BoNT‐A injections to the bladder neck may be an effective therapeutic option in addressing pain, LUTS, and elevated urine volumes in women with BNO.

In the neurogenic population, BoNT‐A has been injected via a transurethral approach into the external urethral sphincter (EUS) for treatment of detrusor sphincter dyssynergia (DSD) with mixed results. In neurogenic patients, some have demonstrated that BoNT‐A to the EUS improves voiding pressures, flow rates, and PVR volumes [19, 20, 21, 22, 23], whereas others have demonstrated no improvement in PVR volumes [24]. BoNT‐A transurethral injections have also demonstrated efficacy in treating female non‐neurogenic dysfunctional voiding [25]. Limited studies have investigated the therapeutic responses of BoNT‐A injections for BNO. A retrospective analysis of 10 female patients with BNO undergoing bladder BoNT‐A demonstrated 70% subjective improvement in symptoms [10]. Pradhan injected BoNT‐A into the bladder neck in 7 female patients with BNO and demonstrated a reduction in PVR by 62% [26]. We similarly report a 68.2% improvement in subjective symptoms post‐procedure and a significant reduction in PVR volumes from a median of 126 mL pre‐procedure to 28 mL post‐procedure (*p* < 0.001). We previously published a retrospective case series of 18 patients receiving BoNT‐A for treatment of female BNO and found that BoNT‐A to the bladder neck significantly improved pelvic pain and refractory hesitancy [11]. The current study prospectively analyzes the therapeutic effect of bladder neck BoNT‐A for females with BNO using validated measures.

Transurethral bladder neck incision (BNI) remains the historical reference standard for female BNO, with multiple series reporting high rates of symptom relief and improved voiding parameters [27, 28, 29]. However, these benefits must be balanced against procedure‐related adverse safety events (ASEs), most notably de novo SUI requiring subsequent continence procedures, along with rare but serious complications such as fistula formation [8, 9]. In contrast, onabotulinumtoxinA (BoNT‐A) injection into the BN offers a tissue‐sparing, reversible intervention that can be repeated, titrated, and discontinued. In women for whom even a modest risk of de novo SUI is unacceptable (e.g., high‐impact lifestyle, occupational constraints, or future pregnancy planning), in those with overlapping pelvic floor hypertonicity or pelvic pain where an irreversible incision may not address the functional component, or in patients with medical comorbidities and anesthesia risk, bladder neck BoNT‐A may serve as a pragmatic first‐line therapy.

The strengths of this study include that we prospectively evaluated patient‐reported outcomes regarding the effect of bladder neck BoNT‐A on treatment of BNO in females. We used validated surveys to evaluate changes in pain, LUTS, and quality of life. The generalizability of this research is limited by the small sample size, the subspecialty referral population, and that patients were recruited from a single academic institution and surgeon. Urodynamic parameters can be heterogeneous, given difficulty relaxing the bladder neck to void in the testing environment, association with autonomic dysfunction in some patients (syncope during testing), and pain. Although all patients received BoNT‐A to the bladder neck, 16 patients received BoNT‐A in other locations; patients were instructed to try to distinguish the benefits separately, but the multiple interventions may confound our results. These patients received BoNT‐A injections to the pelvic floor and the bladder neck to address concomitant high‐tone pelvic floor dysfunction (HTPFD). HTPFD is frequently present in this population and can exacerbate LUTS. While this reflects real‐world overlap of pelvic floor and bladder outlet pathology, we recognize the potential confounding as improvements may not be attributable exclusively to BoNT‐A to the bladder neck. An additional limitation is that although we collected the PVR pre‐ and post‐procedure, we did not collect data on Qmax post. Collecting this information would have improved our results. Nevertheless, BNO in women is a rare diagnosis, and we present prospective data for 22 patients with follow‐up, with a 100% response rate. A randomized controlled trial comparing BoNT‐A injections to the bladder neck versus a comparator would be a helpful next step in determining efficacy.

## 5 Conclusion

BNO in women is a lower urinary tract condition with serious quality of life implications and limited therapeutic options. BoNT‐A to the bladder neck demonstrated significant improvement in patients' pain, LUTS, and PVR volumes. Larger, multi‐institutional, randomized studies are needed to determine the difference in outcomes of BoNT‐A to the bladder neck versus placebo or active control for BNO in women.

## Author Contributions

Elise J. B. De and Brittany Lee Roberts conceived and designed project. Elise J. B. De, and Darrell Bibicheff acquired the data. Elise J. B. De and Brittany Lee Roberts analyzed and interpreted the data. Elise J. B. De, Darrell Bibicheff, and Brittany Lee Roberts wrote the paper and provided editing.

## Funding

The authors received no specific funding for this work.

## Ethics Statement

Ethics approval was obtained by the Albany Medical College Institutional Review Board prior to initiation of this study.

## Consent

Patients provided written informed consent.

## Conflicts of Interest

The authors declare no conflicts of interest.

## Data Availability

The data that support the findings of this study are available on request from the senior author, EJBD, upon reasonable request.
